# A retrospective cohort study of the effect of sugammadex versus neostigmine on postoperative gastrointestinal motility in open colorectal surgical procedures

**DOI:** 10.1016/j.sipas.2023.100233

**Published:** 2023-12-24

**Authors:** Taylor N. Harris, Eric G. Johnson, Aric Schadler, Jitesh Patel, Ekaterina Fain, Laura M. Ebbitt

**Affiliations:** a Department of Pharmacy, University of Kentucky HealthCare, Lexington, KY, USA; b University of Kentucky College of Pharmacy, Lexington, KY, USA; c Department of Anesthesiology, University of Kentucky HealthCare, Lexington, KY, USA; d Division of Colorectal Surgery, Department of Surgery, University of Kentucky College of Medicine, Lexington, KY, USA

**Keywords:** Colon surgery, Anesthesia, Sugammadex, Neostigmine, Post-operative ileus

## Abstract

**Introduction:**

Neuromuscular blockers (NMB) are used in surgical procedures to facilitate muscle relaxation and intubation. NMBs are then reversed at the end of the surgery with either an acetylcholinesterase inhibitor, such as neostigmine, or a modified cyclodextrin compound, such as sugammadex. Neostigmine and glycopyrrolate elicit counteracting cholinergic effects, potentially impairing postoperative gastrointestinal motility. This may have higher significance in colorectal surgery procedures given the baseline risk of delayed postoperative motility associated with some operations.

**Methods:**

This is a retrospective, single-center, cohort review of open colorectal procedures that received either sugammadex alone, or neostigmine with glycopyrrolate. The primary outcome was time from end of colorectal procedure to time of first bowel movement. Secondary outcomes included incidence of postoperatively placed nasogastric tubes, nausea and vomiting, need for motility agents or TPN, hospital length of stay, 30-day hospital readmission or return to emergency department (ED).

**Results:**

A total of 99 patients were included in the sugammadex group and 350 in the neostigmine + glycopyrrolate group. Time to first bowel movement was significantly faster in the sugammadex group (61.7 h) compared to the neostigmine group (71.9 h) (*p* = 0.03). Secondary outcomes were similar between the two groups, except for a higher incidence of postoperative nausea and vomiting in the sugammadex group (*p* = 0.04).

**Discussion:**

These findings support that sugammadex may be utilized for open colorectal procedures to aid in earlier discharge which could lead to differences in length of stay. Using sugammadex did not result in patients requiring additional interventions or experiencing symptoms of post-operative ileus.

**Conclusion:**

Compared to neostigmine and glycopyrrolate, patients receiving sugammadex for neuromuscular blockade reversal in open colorectal procedures experienced a shorter time to return of bowel function. The results from this study confirm the findings from previous retrospective studies that were conducted.

## Highlights

• Sugammadex demonstrated faster return of bowel function for open colorectal surgeries.
• The sugammadex group experienced more postoperative nausea and vomiting.
• No differences in length of stay, need for TPN, readmission, or motility agents between groups.

## Introduction

Postoperative ileus (POI) is a common complication following all abdominal surgery and has a reported overall incidence of up to 30 % [1]. Within colorectal surgery rates of ileus are similar, with as many as 14.3 % of patients also experiencing prolonged POI, delaying discharge [2,3]. POI negatively impacts both patients and healthcare institutions and is associated with increases in patient morbidity, health care costs, and hospital length of stay [4]. The cause of POI is multifactorial and may be influenced by: surgical approach, duration, bowel handling, American Society of Anesthesiologists (ASA) score, anesthesia, fluid administration, transfusion needs, opioid use, and delayed postoperative mobilization [4], [5], [6]. In addition to these risk factors, neuromuscular blockade and reversal strategy utilized during the surgical procedure may also influence the prompt recovery of postoperative bowel function.

Non-depolarizing neuromuscular blockers (NMBs) such as rocuronium and vecuronium are commonly used during surgical procedures to facilitate endotracheal intubation and inhibit muscle contraction during the surgical procedure [7]. This is accomplished through competitively inhibiting the binding of acetylcholine to post-synaptic nicotinic receptors within the neuromuscular junction of the motor plate to prevent depolarization that [8]. NMB reversal agents, such as neostigmine and sugammadex, are often used at the conclusion of surgical procedures to reverse the effects of remaining NMB agents. Neostigmine is an acetylcholinesterase inhibitor which increases acetylcholine concentrations at the neuromuscular junction, facilitating competition with NMB agents at the post-synaptic acetylcholine receptors. The effect of increased concentrations of acetylcholine is not limited to the neuromuscular junction, and excessive acetylcholine may bind to M_2_ receptors on cardiac cells, facilitating bradycardia through enhanced parasympathetic tone. To mitigate the potential parasympathetic effects, neostigmine is co-administered with an antimuscarinic, typically glycopyrrolate or atropine [9]. By antagonizing muscarinic receptor activity, glycopyrrolate and atropine have the potential to impair gastric motility by decreasing motility of smooth muscles, including the gastrointestinal tract. Conversely, sugammadex forms a strong covalent bond with rocuronium or vecuronium, eliminating their potential to bind to nicotinic acetylcholine receptors [10]. As the action of sugammadex does not have a direct effect on acetylcholine receptors, co-administration of an anticholinergic is not required.

A prior systematic review showed that sugammadex was 6.6 times faster at reversing moderate neuromuscular blockade compared to neostigmine and was associated with 40 % fewer adverse effects [11]. Prior studies have found that neostigmine can increase gastrointestinal motility following colon surgery; however, data is still emerging comparing the efficacy of sugammadex. A recent retrospective study demonstrated that the use of sugammadex resulted in faster return of bowel function by 11.7 h (*p* = 0.004) than neostigmine with glycopyrrolate following laparoscopic colorectal surgery [12]. However, it is not known whether there is a difference in return of bowel function between neostigmine and sugammadex in patients who undergo open colorectal surgical procedures. Prior studies have shown that patients who undergo laparoscopic colorectal surgery have faster recovery of bowel function than patients who undergo open colorectal surgery [5,13]. This study was completed to determine the time to first bowel movement and incidence of POI in this high-risk population.

## Materials and methods

### Study design and study population

This was an observational, retrospective cohort study that took place at a single-center academic medical center done in accordance with the STROCSS 2021 guidelines [14]. Patients were included in this study if they were ≥18 years old and received an open colectomy between January 1, 2016 and June 1, 2021. Patients had to receive a non-depolarizing aminosteroidal neuromuscular blocker (vecuronium, rocuronium) for intraoperative maintenance as well as either sugammadex or neostigmine as the NMB reversal agent. Open colorectal surgical procedures were identified through surgical Current Procedural Terminology (CPT) codes 44140–44160. Patients were allowed to receive concomitant surgeries during their colorectal procedure. Patients were excluded from the study if they underwent a laparoscopic surgery, received both neostigmine and sugammadex as NMB reversal agents, had re-operations during the same hospital admission, if they were listed as having an emergent colorectal procedure that was to be performed within one hour of posting, did not require admission postoperatively, or were pregnant. Data points were collected from the patient's medical record using the institution's electronic health record. The Center for Clinical and Translational Science, a data collection team at UK HealthCare, assisted with identification of study patients and extraction of data from the electronic health records (grant number UL1TR001998). The study protocol was approved by the University of Kentucky Institutional Review Board as an exempt protocol and was carried out in accordance with the Code of Ethics of the World Medical Association. Informed consent was not required given the retrospective and low-risk nature of the study.

### Variables of interest and outcomes

The primary endpoint was time, in hours, to first charted bowel movement. This was defined as the length of time between the end of the colorectal surgery and the first postoperative bowel movement recorded in the electronic medical record. Bowel movements were defined as ≥50 mL of liquid stool through an ileostomy or colostomy or a stool bowel movement. Flatulence was not recorded due to inconsistent documentation. In the absence of a documented bowel movement prior to discharge, event time was assigned as 12:00 pm (noon) on the day of hospital discharge. Secondary endpoints included placement of nasogastric tubes, documentation of postoperative nausea and vomiting, use of postoperative motility agents, use of postoperative total parenteral nutrition (TPN), hospital length of stay, readmission within 30 days of hospital discharge, and return to the emergency department (ED) within 30 days of hospital discharge. Postoperative nausea and vomiting was defined as >2 doses of an “as needed” anti-emetic per day or a recorded episode of emesis. Motility agents included metoclopramide, erythromycin, azithromycin, or subcutaneous neostigmine.

### Statistical analysis

Categorical variables were reported as counts and percentage of the population and continuous variables were reported at median (interquartile range [IQR]). Levene's Test for Equality of Variances was used to determine normality of data sets. Student *t*-tests and Mann–Whitney *U* tests (for unevenly distributed data) were used to assess continuous variables and Chi-square and Fischer's exact tests (for unevenly distributed data) were used to assess categorical variables. Multiple regression was performed on the primary outcome utilizing a backwards elimination variable selection criterion to account for potential confounding variables. Statistically significant variables were used for the multivariate analysis and included postoperative nausea and vomiting and choice of neuromuscular blocker reversal agent.

Based on prior studies evaluating return of bowel function between colorectal surgery patients who received neostigmine versus sugammadex, we proposed an effect size between the 2 treatment groups of 12 hours [15]. This resulted in a need for *n* = 99 patients in the sugammadex group and *n* = 100 patients in the neostigmine group to reach statistical power of 80 %. SAS 9.4 and SPSS 28.0 (SPSS Inc., Armonk, NY, USA) were used to perform all statistical analyses.

## Results

### Patient characteristics

**Fig. 1:**
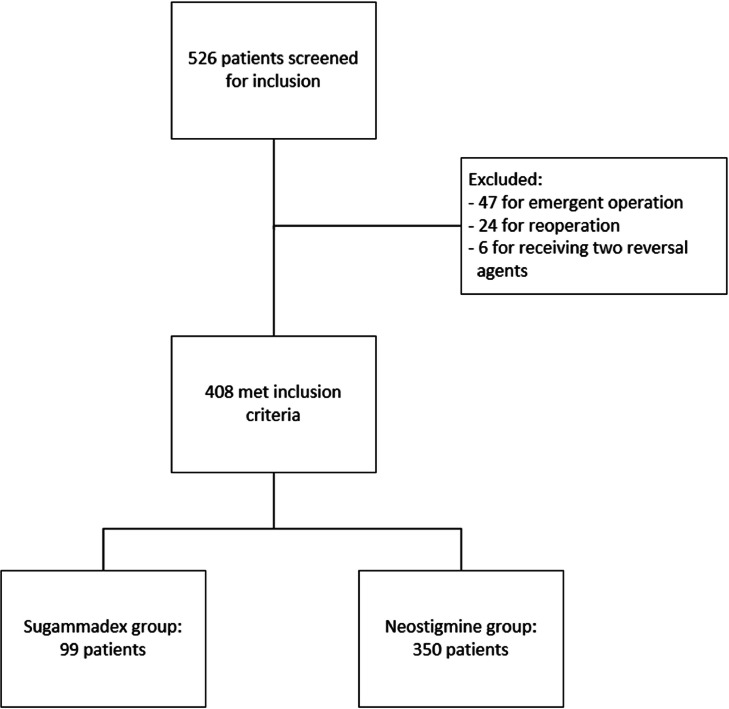
Study flow diagram.

A total of 526 patients were screened, with 77 patients meeting exclusion criteria (Fig. 1). Ultimately, 408 patients were included in the study with 99 patients in the sugammadex group and 350 patients in the neostigmine group. Patient demographics are shown in Table 1. Patient demographics were similar between the treatment groups except for a longer procedure time in the sugammadex group (median 4.4 h in the sugammadex group, 3.8 h in the neostigmine group; *p* = 0.015). The groups were similar in characteristics with a median age was 57 years old, with a majority of patients identifying as Caucasian and female. The most common indication for colon surgery was cancer, that occurred in almost two-thirds of patients. 77.8 % of patients underwent a partial colectomy in the sugammadex group as compared with 84.6 % in the neostigmine group. Concomitant procedures were performed in 25.2 % of patients in the sugammadex group and 30.6 % of patients in the neostigmine group. The most common concomitant procedures performed were total abdominal hysterectomy with bilateral salpingo-oophorectomy (TAH BSO), hyperthermic intraperitoneal chemotherapy (HIPEC), and cholecystectomy

**Table 1:** Patient demographics.

	Sugammadex n=99	Neostigmine *n* = 350	*p*-value
Age, years	57.2 ± 14.0	57.7 ± 14.3	0.75
Male sex	42 (42.4)	142 (40.6)	0.74
Caucasian race	94 (93.5)	326 (93.1)	0.52
Median BMI	28.5 [24.0–35.6]	27.1 [23.4–32.3]	0.13
Procedure length, h	4.4 [3.1–6.4]	3.8 [2.8–5.1]	0.02
Emergent procedure, #	8 (8.1)	34 (9.7)	0.17
ASA score			0.36
I or II	14 (14.1)	80 (22.9)	
III	78 (78.8)	244 (69.7)	
IV	5 (5.1)	17 (4.9)	
Intraoperative transfusion	12 (12.1)	31 (8.9)	0.33
Indication for surgery			
Cancer	65 (65.7)	205 (58.7)	0.21
Diverticulitis	17 (17.2)	64 (18.3)	0.80
Crohn's disease	9 (9.1)	31 (8.9)	0.94
Ulcerative colitis	1 (1.0)	3 (0.9)	1.00
Additional procedure performed during colorectal procedure	25 (25.2)	107 (30.6)	0.13
Epidural	59 (59.6)	201 (57.4)	0.70

*values reported as mean ± standard deviation, median [IQR], or number (%)

### Primary outcome

**Fig. 2:**
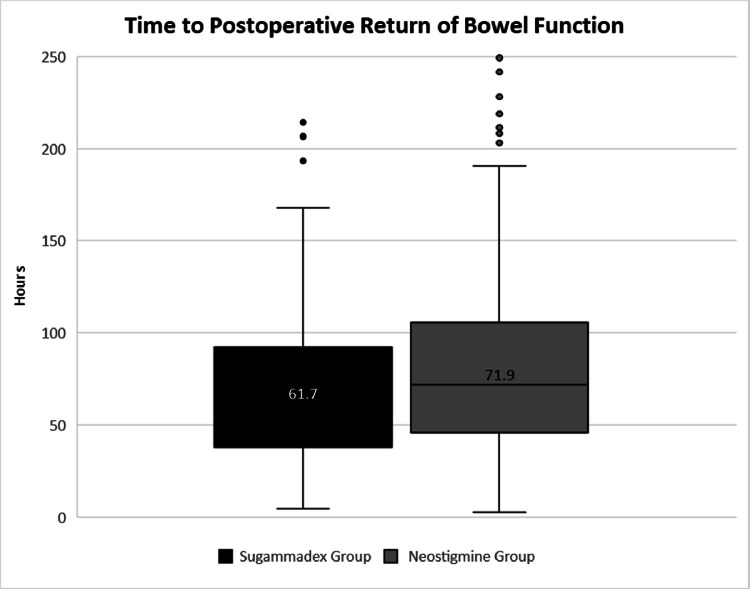
Primary outcome.

Return of bowel function occurred at a median 61.7 h [IQR 38.0 h – 92.2 h] in the sugammadex group and 71.9 h [IQR 45.8 h – 105.5 h] in the neostigmine group (p = 0.03) (Fig. 2). The primary outcome remained statistically significant after applying a multivariate analysis that included type of NMB reversal agent, incidence of postoperative nausea and vomiting, if cases were elective, procedure length, and patients BMI (*p* = 0.02). With regard to the multivariate analysis, procedure length was time was removed first (*p* = 0.87), then BMI (*p* = 0.43), then emergent procedure (*p* = 0.15), which resulted in the neuromuscular blocker and postoperative nausea and vomiting being significant with *p*-values of 0.02 for both.

### Secondary outcomes

There were no differences in hospital length of stay (*p* = 0.90), need for postoperative TPN (*p* = 0.15), nasogastric tube placed postoperatively (*p* = 0.82), readmission within 30 days of discharge (*p* = 0.08), return to the emergency department within 30 days of discharge (*p* = 0.42), or need for postoperative motility agent (*p* = 0.67) between the sugammadex and neostigmine groups (Table 2). The incidence of postoperative nausea and vomiting was higher in the sugammadex group (36.4 %) compared to the neostigmine group (26.0 %, *p* = 0.04).

**Table 2:** Secondary outcomes.

Secondary Outcomes	Sugammadex n = 99	Neostigmine n = 350	*p* value
Hospital length of stay (days)	7.5 [5.9–11.3]	7.7 [5.5–11.0]	0.90
Readmitted within 30 days	12 (12.1)	69 (19.7)	0.08
Return to ED within 30 days	6 (6.1)	30 (8.6)	0.42
TPN postoperatively	7 (7.1)	13 (3.7)	0.15
NG tube placed postoperatively	16 (16.2)	60 (17.1)	0.82
Postoperative nausea/vomiting	36 (36.4)	91 (26.0)	0.04
Motility agent used postoperatively	16 (16.2)	63 (18.0)	0.67

*values reported as median [IQR] or number (%)

## Discussion

In this retrospective, single-center, cohort study, sugammadex was associated with a faster return of bowel function compared to neostigmine in patients receiving an open, colorectal surgery. This is despite a longer length of procedure time in the sugammadex group. The absolute difference in time to return of bowel function between the sugammadex and neostigmine groups was 10 hours, which could be considered clinically significant due to potentially being discharged from the hospital sooner. It is important to note that the American Society of Anesthesiologists 2023 Practice Guidelines also recommends sugammadex over neostigmine when moderate to deep neuromuscular blockade is induced by rocuronium or vecuronium, although these recommendations are not specific to colon surgeries [7].

The findings from this study are consistent with those of prior studies examining the time to return of bowel function between sugammadex and neostigmine in colorectal surgery patients. A similar study was conducted by Hunt and colleagues which found a return of bowel function at 41.7 hours in the sugammadex group and 53.4 hours in the neostigmine group, for an absolute time difference of 11.7 hours [12]. Although the absolute time difference is similar between the Hunt et al study and our study, the time to return of bowel function in both groups was faster in the Hunt study compared to our study. In our study, the time to return of bowel function was 61.7 hours in the sugammadex group and 71.9 hours in the neostigmine group. The longer time to return of bowel function could be explained our medically complex patients that received concomitant operations during their colorectal procedure as well as longer OR times. In addition, we specifically studied open colorectal procedures as opposed to only laparoscopic in the Hunt study, and that patients undergoing open colorectal procedures have a longer return of bowel function compared to laparoscopic procedures. Similarly, Traeger and colleagues compared sugammadex and neostigmine in a mixed population of open and laparoscopic major bowel surgery procedures [15]. The results from the Traeger study are in agreement with both our study and the Hunt et al study. The authors found that sugammadex was associated with a faster gastrointestinal recovery time, defined as the time to first stool and tolerance of solid diet without significant nausea or vomiting. It is important to note that there were more patients in the sugammadex group who received a laparoscopic procedure (66.7 %) compared to patients in the neostigmine group (50.9 %) (*p* = 0.006) which could result in neostigmine having a longer return to bowel function. Finally, a meta-analysis of five studies was performed and found that colorectal surgical patients receiving sugammadex experienced faster return of bowel function (*p* ≤ 0.0001) without differences in length of stay [16]. Postoperative ileus was reported in 4 of the included studies and the pooled analysis showed no significant different in postoperative ileus rates (OR 1.44, *p* = 0.97).

Our study did not find a statistically significant difference between groups for the following secondary endpoints: hospital length of stay, 30-day readmission, return to ED within 30 days, postoperative TPN, postoperative NG tube insertion, and use of postoperative motility agent. Our study found a statistically higher incidence of postoperative nausea and vomiting in the sugammadex group which is not in agreement with the results from the Hunt et al study which found a lower incidence of postoperative nausea and vomiting in the sugammadex group. It is unclear if this is a chance finding that is clinically significant; nonetheless, the increased incidence of postoperative nausea and vomiting in the sugammadex group did not result in an increase in the number of NG tubes placed postoperatively, need for TPN postoperatively, or time to return to bowel function in our study. Additionally, this study was not set up to evaluate if patients received appropriate PONV prophylaxis, but should be considered in future studies for further analysis. This study was not able to evaluate why patients experienced a quicker return of bowel function when sugammadex was utilized, but this did not translate into a short length of stay or decreased need for motility agents. Therefore, that is an area in which future studies could potentially focus.

Our study has several strengths. First, we included a medically complex patient population with many patients receiving concomitant operative procedures, representative of real-life practice. We also addressed the gap of literature surrounding the use of sugammadex and neostigmine in patients receiving open colorectal procedures as opposed to laparoscopic and were powered to detect a difference. We acknowledge that our study has several limitations. Given the retrospective nature of our study, we relied on accurate and complete documentation in the electronic health record for our data. Certain data points were not collected, such as opioid administrations and patient controlled analgesia (PCA) doses along with chronic pain as a baseline demographic, which may have had an impact on return of bowel function. Additionally, despite a diverse disease state population, our study lacked racial diversity with approximately 93 % of patients being Caucasian, which may limit extrapolation of results to other ethnicities.

## Conclusions

In conclusion, our study found a faster return of bowel function in patients receiving sugammadex versus neostigmine during open, colorectal procedures. These results confirm the findings from previous studies examining the use of sugammadex versus neostigmine in colorectal surgery patients.

## CRediT authorship contribution statement

**Taylor N. Harris:** Data curation, Investigation, Methodology, Writing – original draft, Writing – review & editing. **Eric G. Johnson:** Conceptualization, Methodology, Project administration, Writing – original draft, Writing – review & editing. **Aric Schadler:** Formal analysis, Methodology. **Jitesh Patel:** Conceptualization, Methodology, Writing – review & editing. **Ekaterina Fain:** Conceptualization, Methodology, Writing – review & editing. **Laura M. Ebbitt:** Conceptualization, Investigation, Methodology, Supervision, Writing – original draft, Writing – review & editing.

## Declaration of Competing Interest

All authors declare that they have no known competing financial interests or personal relationships that could have appeared to influence the work reported in this paper.
